# Development and Validation of a Novel Survival Model for Cutaneous Melanoma Based on Necroptosis-Related Genes

**DOI:** 10.3389/fonc.2022.852803

**Published:** 2022-03-21

**Authors:** Zehao Niu, Xin Wang, Yujian Xu, Yan Li, Xiaojing Gong, Quan Zeng, Biao Zhang, Jiafei Xi, Xuetao Pei, Wen Yue, Yan Han

**Affiliations:** ^1^ Medical School of Chinese PLA, Beijing, China; ^2^ Department of Plastic and Reconstructive Surgery, The First Medical Center, Chinese PLA General Hospital, Beijing, China; ^3^ Department of Ophthalmology, PLA Strategic Support Force Characteristic Medical Center, Beijing, China; ^4^ Stem Cell and Regenerative Medicine Lab, Institute of Health Service and Transfusion Medicine, AMMS, Beijing, China; ^5^ South China Research Center for Stem Cell and Regenerative Medicine, SCIB, Guangzhou, China; ^6^ Academy of Military Medical Sciences (AMMS), Academy of Military Sciences, Beijing, China

**Keywords:** melanoma, necroptosis, TCGA, prognostic prediction, treatment, biomarkers

## Abstract

**Background:**

Necroptosis is crucial for organismal development and pathogenesis. To date, the role of necroptosis in skin cutaneous melanoma (SKCM) is yet unveiled. In addition, the part of melanin pigmentation was largely neglected in the bioinformatic analysis. In this study, we aimed to construct a novel prognostic model based on necroptosis-related genes and analysis the pigmentation phenotype of patients to provide clinically actionable information for SKCM patients.

**Methods:**

We downloaded the SKCM data from the TCGA and GEO databases in this study and identified the differently expressed and prognostic necroptosis-related genes. Patients’ pigmentation phenotype was evaluated by the GSVA method. Then, using Lasso and Cox regression analysis, a novel prognostic model was constructed based on the intersected genes. The risk score was calculated and the patients were divided into two groups. The survival differences between the two groups were compared using Kaplan-Meier analysis. The ROC analysis was performed and the area under curves was calculated to evaluate the prediction performances of the model. Then, the GO, KEGG and GSEA analyses were performed to elucidate the underlying mechanisms. Differences in the tumor microenvironment, patients’ response to immune checkpoint inhibitors (ICIs) and pigmentation phenotype were analyzed. In order to validate the mRNA expression levels of the selected genes, quantitative real-time PCR (qRT-PCR) was performed.

**Results:**

Altogether, a novel prognostic model based on four genes (BOK, CD14, CYLD and FASLG) was constructed, and patients were classified into high and low-risk groups based on the median risk score. Low-risk group patients showed better survival status. The model showed high accuracy in the training and the validation cohort. Pathway and functional enrichment analysis indicated that immune-related pathways were differently activated in the two groups. In addition, immune cells infiltration patterns and sensitivity of ICIs showed a significant difference between patients from two risk groups. The pigmentation score was positively related to the risk score in pigmentation phenotype analysis.

**Conclusion:**

In conclusion, this study established a novel prognostic model based on necroptosis-related genes and revealed the possible connections between necroptosis and melanin pigmentation. It is expected to provide a reference for clinical treatment.

## Introduction

Skin cutaneous melanoma (SKCM) derives from melanocytes and is usually secondary to sun exposure (1). The death toll of SKCM was approximately 55,500 per year, and the incidence of cutaneous melanoma is still rising at a faster rate than other skin cancers (2). In 2019, it became the third most prevalent cancer among males (684,470 cases) and the fifth among females (672,140 cases) (3). Even though the progress of chemotherapy and immunotherapy have greatly improved the treatment and survival status of SKCM patients, 5-year survival of stage IV SKCM is no more than 19% (3, 4). At present, diagnoses for melanoma patients are still based on histopathological examination, which is not conducive to early detection and interventions (5). This may account for treatment delay and high mortality. During these years, individual-specific therapies have become increasingly important. Thus, it is essential to explore prognostic targets and models.

Necroptosis is a kind of regulated necrosis triggered by cell stress, damage or infection. Necroptosis shares similar morphological features with necrosis, such as cell membrane rupture, translucent cytoplasm and swelling of organelles (6). As illustrated in previous literature, necroptosis is both a friend and foe in tumor progression (7). On the one hand, it could work as a complementary form of programmed cell death if cancer cells escape from apoptosis. On the other hand, necroptosis may result in the activation of the inflammatory response, which could induce tumor progression (8). Triggers of necroptosis include RIPK1/RIPK3 and MLKL (9). When the cell death model is swished into necroptosis, activated RIPK1/3 could induce the formation of necrosome, which could cause the rapture of the cell membrane. Then, MLKL was activated as the downstream reaction in necrosomes. MLKL is translocated to the plasma membrane and executes necroptosis. It was reported that necroptosis is critical for SKCM development and progression. For example, Geserick et al. reported that RIPK3 was down-regulated in melanoma tissues. Furthermore, decreased RIPK3 predicted a worse prognosis in SKCM (10). Thus, therapeutic methods targeting necroptosis have attracted significant attention (11, 12).

In general, the single-gene expression usually has limited prognostic implications. Thus, prognostic models based on a cluster of genes associated with specific characters of tumors have gained lots of attention. These models were based on metastasis-related genes (13), immune-related genes (14), or IFNγ response-related genes (15). To date, no study has fully illustrated the role of necroptosis-related genes in prognosis prediction of cutaneous melanoma. Moreover, many studies based on the bioinformatic method have neglected melanin pigmentation, which plays an essential role in melanoma biology (16, 17). Thus, this study aimed to develop a novel prognostic prediction model based on necroptosis-related genes and analyze the pigmentation phenotypes to provide helpful information for clinical decision-making.

## Materials and Methods

### Data Acquisition and Normalization

We started by downloading the RNA sequencing data (FPKM form) and corresponding clinical data of each SKCM patient from the TCGA website. Patients without complete clinical data and patients with survival <90 days were excluded. Finally, 443 patients were eligible for this study. RNA sequencing data (FPKM, log_2_(x+0.001) transformed) of 813 normal skin samples (including sun-exposed and not sun-exposed skin samples) were acquired from the UCSC Xena website. GSE65904 (based on GPL10558, including 214 patients) was obtained from the GEO database. Patients without complete survival data were eliminated. Finally, 210 patients were included in this study. The necroptosis signature (n = 65) was downloaded from the Gene Set Enrichment Analysis (GSEA) database (18).

### Identification of Differently Expressed and Prognosis Related Genes

We extracted 65 necroptosis-related genes from the RNA sequencing data of TCGA and GTEx datasets. Skin samples in GTEx were used as a normal control for TCGA tumor samples (19). Then, the differently expressed genes (DEGs) between normal and tumor samples were identified (“limma” package). The cutoffs were set as *P*<0.05 and log|(fold change)|>0.586. Then, using the “limma” package, the RNA sequencing data and clinical data acquired were combined. Univariate Cox regressions were performed to identify the prognosis-related genes, and the cutoff was set as *P*<0.05. Intersected genes were chosen as candidate genes, and their expression correlation networks were visualized using the “igraph” package.

### Construction of Prognostic Gene Signature and Nomogram Construction

Firstly, to minimize overfitting and find the most important markers, the LASSO regression was performed. Secondly, a model was built using a stepwise multivariate Cox regression analysis. The risk score was calculated by multiplying the coefficients derived from multivariate Cox regression analysis with expression data of each gene. The median risk score was used to divide the SKCM patients into the high-risk group and the low-risk groups. The area under the ROC curve was used to evaluate the predictive performance of this model. The Kaplan-Meier (K-M) survival curve was used to assess patients’ survival status. Then, the prognostic factors were comprised in the construction of the nomogram, and the predictive accuracy was evaluated by the area under the ROC curve.

### Validation of Prognostic Gene Signature

The GSE65904 dataset (external validation cohort) was used to confirm the model’s prognostic value. The risk scores of patients were generated using the same formula. ROC analysis and survival curve analysis were performed as aforementioned.

### Function and Pathway Enrichment Analysis

Firstly, the differently expressed genes (DEGs) between the two groups were identified, and the threshold was set as *P*<0.05 and log|(fold change)|>1. Gene Oncology (GO) and Kyoto Encylopedia of Genes and Genomes (KEGG) analyzes were used to investigate the potential functions and pathways of these DEGs by using the “clusterprofile” package. The gene set enrichment analyses (GSEA) (20) were then used to further identify the enriched pathways in low- and high-risk groups using GSEA software.

### Evaluation of Tumor Microenvironment

The association between the tumor microenvironment and the risk score was investigated further. Using the “ESTIMATE” package, the immune score, stromal score, and tumor purity were calculated (21). The violin plot was used to show the differences between the two groups. Furthermore, the differences in immune cell and immunological pathways were assessed using the ssGSEA method (22).

### Prediction of Patient Response to ICIs and Evaluation of Checkpoint Molecules

Patient response to immune checkpoint inhibitors (ICIs) was predicted by immunophenoscore (IPS) obtained from https://tcia.at/ (23). Checkpoint molecules related to treatment response to ICIs were obtained from previous literature. The expression of these genes was visualized in a box plot.

### Calculation of the Pigmentation Score and Evaluation of Pigmentation Phenotype

As is well illustrated by previous literature, melanin pigmentation plays a vital role in melanoma biology, including immunology, vascularization, cell cycling and tumor environment. Thus, the pigmentation phenotype was taken into consideration in this current study. The pigmentation score was calculated based on pigmentation-related genes (downloaded from the GSEA database) and a gene set variation analysis (GSVA) (24). GSVA is a GSE method that could estimate the pathway’s activation in an unsupervised manner (25). The GSVA score was recognized as the pigmentation score, which represented the pigmentation status of each patient. Firstly, the RNA sequencing data from the TCGA dataset were transformed from FPKM form to TPM form and merged with the RNA sequencing data from the GEO dataset. Then, the pigmentation of each patient was calculated. Patients were divided into high and low-pigmentation score groups based on the median. The difference in pigmentation scores between TCGA and GEO groups was compared.

### Samples Collection

After being approved by the Ethic Committee of Chinese PLA General Hospital (approval number: S2021-185-01, approval date: 2021.05.18), we collected 16 pairs of melanoma tissue and adjacent normal skin samples. All patients provided written informed consent for the publication and their consent to participate in this study.

### RNA Isolation and qRT-PCR Analysis

The expression levels of the four genes used to generate the prognostic model were validated using the qRT-PCR experiment. TRIzol reagent was used to extract total RNA from melanoma and normal skin samples. Then, RNA was converted into cDNA by reverse transcription, and qRT-PCR was performed with SYBR (Takara, Japan). The 2^-ΔΔCT^ method was used to analyze the data (26). GAPDH was used as an internal reference, and the primers were listed in **Supplementary Table 1**.

### Statistical Analysis

R software was used to conduct all statistical analyses (version 4.0.3). Mean and SD were used to express the continuous data, whereas numbers and percentages were used to express the categorical variables. For categorical variables, theχ^2^ or Fisher’s exact test was performed, while for continuous data, the Student’s t-test or Wilcoxon test was performed. Univariate Cox regressions were used to select genes associated with prognosis. For survival analysis, the Kaplan–Meier method was used, and AUC values were used to evaluate the model’s predictive ability.

## Results

**Figure 1:**
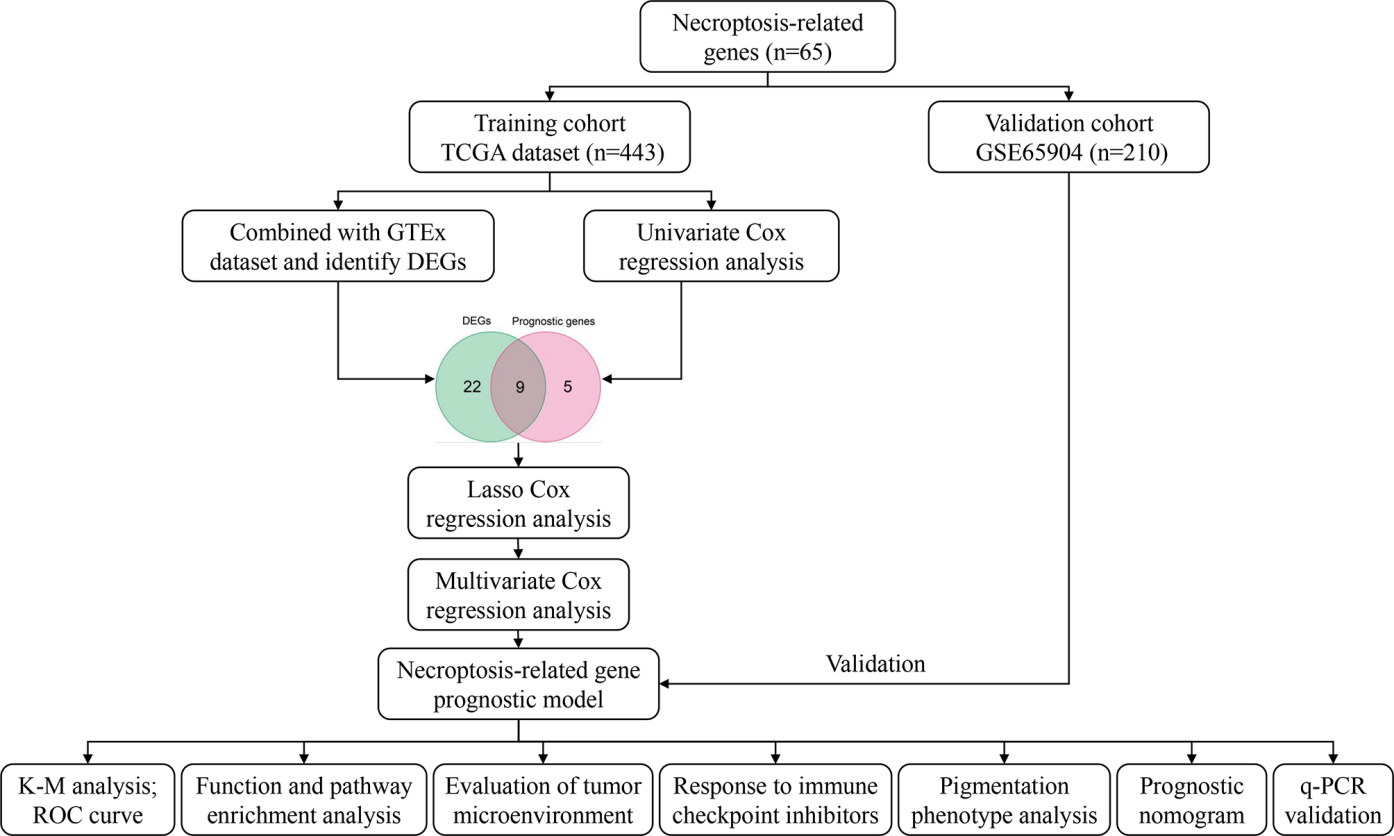
Workflow diagram.

**Figure 1** shows the process of the study.

### Identification of DEGs and Prognostic Genes

**Figure 2:**
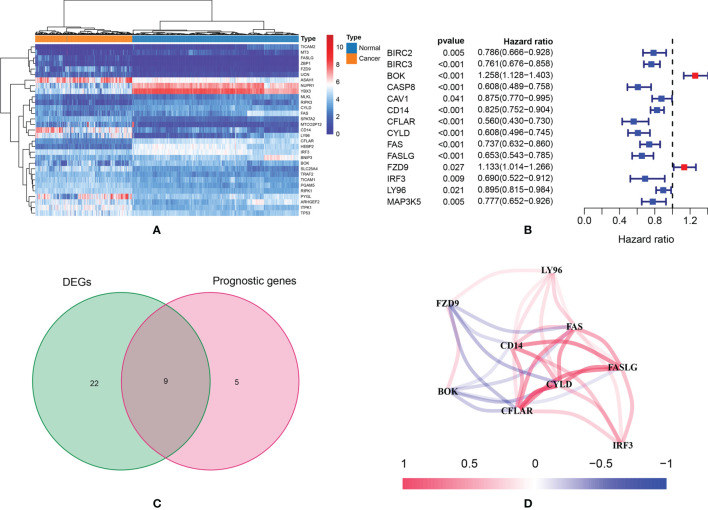
**(A)** The heatmap of necroptosis-related genes which were differently expressed; **(B)** According to the results of univariate Cox regression analysis, a total of 14 genes were identified as prognostic genes; **(C)** Nine intersected genes were selected as target genes; **(D)** Correlation network of intersected genes.

According to the preset thresholds, 31 DEGs were identified (**Figure 2A**). There were 18 up-regulated genes and 13 down-regulated genes. In univariate Cox regressions, 14 prognostic genes were identified (**Figure 2B**). There were 12 protective genes (HR < 1) and two risky genes (HR>1). Finally, nine intersected genes were selected for further studies: BOK, CD14, CFLAR, CYLD, FAS, FASLG, FZD9, IRF3 and LY96 (**Figure 2C**). **Figure 2D** showed the expression correlation network of these intersected genes (positive correlations were marked as red, and negative correlations were marked as blue).

### Development and Validation of Prognostic Model

**Figure 3:**
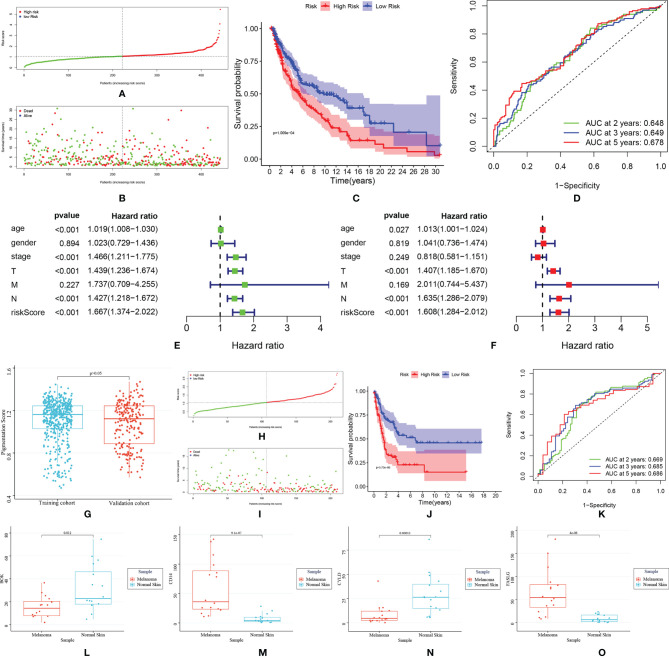
TCGA training cohort: **(A)** Risk score distribution; **(B)** Survival status distribution; **(C)** Kaplan–Meier (KM) curves of overall survival. **(D)** The ROC curves for 2-, 3-, and 5-year survival; **(E, F)** Cox regression analysis of risk score and other clinical features [age, stage, gender, stage-T, stage-N, stage-M); **(D)** The comparison of pigmentation score between training and testing cohort. GEO validation cohort: **(H)** Risk score distribution; **(I)** Survival status distribution; **(J)** Kaplan–Meier (KM) curves of overall survival; **(K)** ROC curve for 2-, 3-, and 5-year survival. **(L–O)**]. The expression of BOK, CD14, CYLD and FASLG in melanoma and normal samples.

In LASSO regression analysis, seven key genes were selected. Then, these genes were included subjected to a stepwise multivariate Cox regression analysis. After excluding non-significant factors one by one in the multivariate analysis, four genes were identified as independent factors for survival (BOK, CD14, CYLD and FASLG). The prognostic model was constructed based on the regression coefficient obtained from the multivariate regression. The risk score was calculated as follows: Risk score = (0.036* BOK expression) +(-0.005* CD14 expression) + (-0.047* CYLD expression) + (-0.097* FASLG expression). Patients in the TCGA cohort were separated into two groups according to their median risk score: high-risk group (n=221) and low-risk group (n=222) (**Figure 3A**). Patients in the low-risk group usually showed better survival (**Figures 3B, C**). The area under the curve (AUC) was 0.648 for two years, 0.649 for three years and 0.678 for five years (**Figure 3D**). According to the results of Cox regression analysis, the risk score could serve as an independent prognostic factor for SKCM patients (**Figures 3E, F**).

The external validation cohort consisted of 210 participants from the GSE65904 dataset. Since pigmentation plays an essential role in melanoma biology, the training and validation cohort’s pigmentation phenotype was first analyzed. The results showed no significant difference in pigmentation score between the two groups, indicating that these groups have similar pigmentation phenotypes and the data from the two groups were comparable (**Figure 3G**). Then, by using the “Scale” package, the expression data were normalized before further studies. The risk score was calculated, and patients were classified into low-risk (n = 99) and high-risk (n = 111). (**Figure 3H**). Similar to the training cohort, the low-risk group patients showed better survival (**Figures 3I, J**). The AUC was 0.669 for two years, 0.685 for three years and 0.686 for five years (**Figure 3K**). The results of qRT-PCR were shown in **Figures 3L–O**. BOK and CYLD were down-regulated, and CD14 and FASLG were up-regulated in melanoma samples, which were in accordance with the bioinformatics results.

### Function and Pathway Enrichment Analysis

**Figure 4:**
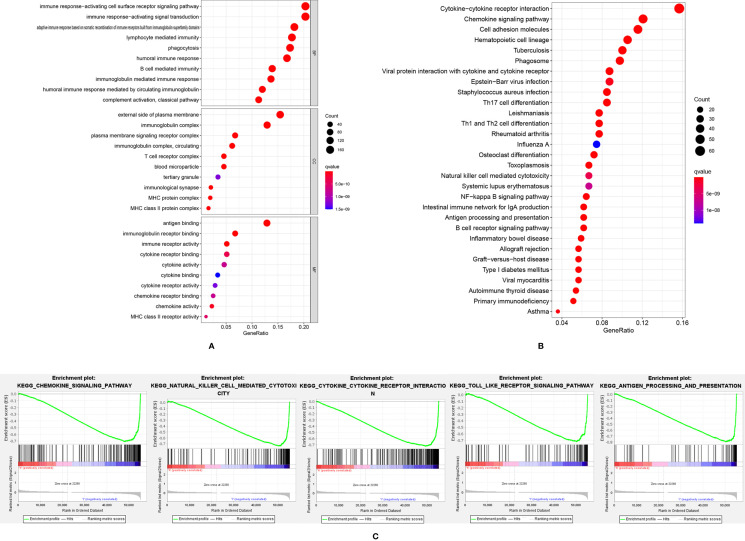
The GO analysis **(A)** and KEGG analysis **(B)** of differently expressed genes; **(C)** GSEA analysis results showed immune-related pathways were highly activated in the low-risk group.

A total of 897 down-regulated and 89 up-regulated genes were identified in the high-risk group. GO and KEGG pathway analysis showed that these differently expressed genes were mainly enriched in immune-related pathways, such as immune response activating cell surface receptor signaling pathway, immune response-activating signal transduction and cytokine-cytokine receptor interactions (**Figures 4A, B**). The GSEA analysis (**Figure 4C**) showed that chemokine signaling pathway, natural killer cell-mediated cytotoxicity, cytokine-cytokine receptor interaction, toll-like receptor signaling pathway and antigen processing and presentation pathway were significantly up-regulated in the low-risk group. The higher level of immune responses in low-risk groups indicated that the tumor microenvironment played an essential role in SKCM formation and progression. Thus, the tumor microenvironment, including immune cells, stromal cells and immune-related pathways were evaluated.

### Evaluation of Tumor Microenvironment

**Figure 5:**
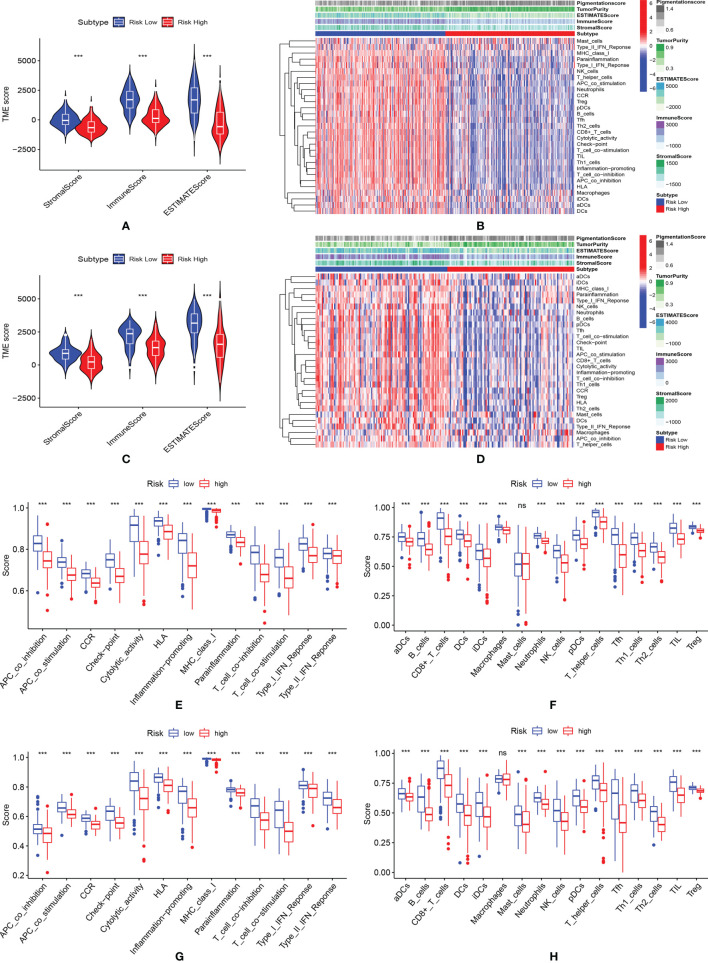
Evaluation of tumor microenvironment. **(A)** The violin plot of the stromal score, immune score and ESTIMATEscore in the training cohort; **(B)** The immunity heatmap of high- and low-risk groups in the training cohort; **(C)** The violin plot of the stromal score, immune score and ESTIMATEscore; **(D)** The Immunity heatmap of high- and low-risk group in the GEO validation cohort; **(E, F)** Comparison of immune-related pathways and infiltration of immune cells between high- and low-risk group in the TCGA cohort; **(G, H)** Comparison of immune-related pathways and infiltration of immune cells between high- and low-risk group in the GEO validation cohort. *** means P < 0.001; ns, not significant.

In both TCGA (**Figure 5A**) and GEO (**Figure 5C**) datasets, the low-risk group showed significantly higher ESTIMATEscore, immune score and stromal score, indicating more immune cells and stromal cells were infiltrated in these samples. Thus, lower tumor purity (**Figures 5B, D**) was shown in these patients. As shown in **Figures 5B, F**, except mast cells, all immune cells were found to be highly enriched in the low-risk group of the TCGA cohort. As shown in **Figures 5D, H**, except for macrophages, all immune cells were found to be highly enriched in the low-risk group of the GEO cohort. In both the TCGA (**Figure 5E**) and the GEO (**Figure 5G**) dataset, the low-risk patient group showed higher activation of immune-related pathways.

### Patient Response to ICIs

**Figure 6:**
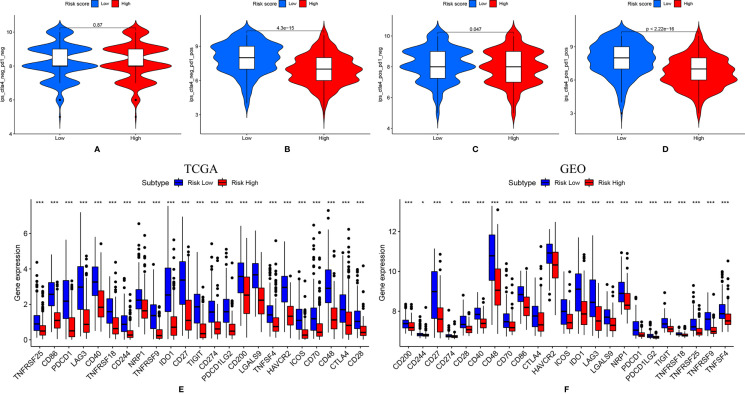
**(A-D)** Patients were more sensitive to immune checkpoint inhibitor when ether PD1 and CTLA-4 was positively expressed; **(E, F)**. Immune checkpoint-related genes were highly expressed in the low-risk group. * means P < 0.05, ** means P < 0.01, *** means P < 0.001.

Patient treatment response to ICIs was shown in **Figures 6A–D**. The scores of IPS with PD1 (programmed cell death protein1) blockers, IPS with CTLA4 (lymphocytes associated protein 4) blockers, IPS with CTLA4 and PD1 blockers were significantly higher in the low-risk patient group, indicating these patients might have better immunotherapy response. The expressions of hub biomarkers of ICIs response were further evaluated in TCGA and GEO dataset (**Figures 6E, F**). In the low-risk group, almost all markers, including PD-1, PD-L1, PD-L2, and CTLA4 were significantly higher expressed.

### Pigmentation Phenotype Assessment

**Figure 7:**
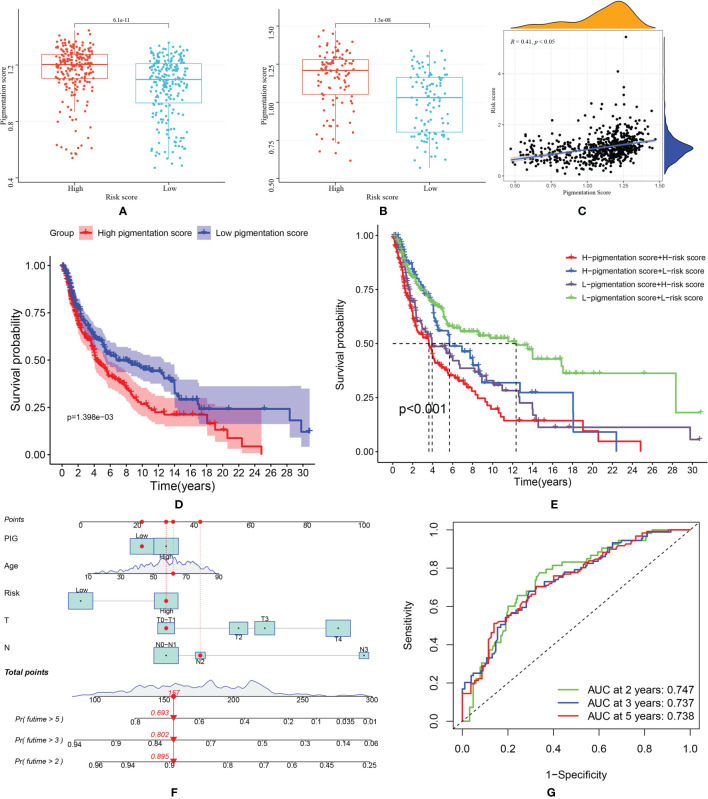
**(A)** Comparison of pigmentation score in high and low-risk score patients from TCGA dataset; **(B)** Comparison of pigmentation score in high and low-risk score patients from GEO dataset; **(C)** Correlation between risk score and pigmentation score; **(D)** Kaplan–Meier (KM) curves of overall survival of high and low- pigmentation score groups; **(E)** The survival curves of SKCM patients based on risk score and pigmentation score; **(F)** The nomogram was constructed based on pigmentation score, age, risk score, T and N stage; **(G)** The ROC curve of nomogram model predicting 2-, 3- and 5-year survival.

Pigmentation score between high and low-risk groups was compared. As shown in **Figures 7A, B**, patients in the high-risk score group tend to have higher pigmentation scores. This trend was observed both in the training and validation group. Then, in the correlation analysis (**Figure 7C**), we found that the risk score was positively related to the pigmentation score (R=0.41, *p*<0.05). Patients were classified into high and low-pigmentation score groups based on the median pigmentation score. In survival analysis, patients in the high-pigmentation score group tend to have worse survival status (**Figure 7D**). If the patients were ranked high in risk and pigmentation scores, they would have a much worse survival (**Figure 7E**). Considering the prediction value of the pigmentation score, a nomogram was constructed based on risk score and other prognostic factors (**Figure 7F**). The AUC was 0.747 for two years, 0.737 for three years and 0.738 for five years, indicating good accuracy in predicting the survival of patients with SKCM (**Figure 7G**).

## Discussion

This study developed a novel prognostic model based on four necroptosis-related genes: BOK, CD14, CYLD and FASLG. In the training cohort, the AUCs of the ROC curve were: 0.648 for two years, 0.649 for three years and 0.678 for five years in the TCGA training cohort and 0.669 for two years, 0.685 for three years and 0.686 for five years in the GEO validation cohort. The GO, KEGG and GSEA analyses revealed that immune-related pathways were highly activated in the low-risk group. Thus, we further evaluated the difference in tumor microenvironment between the two groups. The results revealed that more stromal cells and immune cells were enriched in the low-risk group. Furthermore, low-risk group patients showed highly expressed checkpoint markers and better ICIs response. In addition, the pigmentation phenotype of patients was evaluated by pigmentation score. The results showed that patients with high-risk and high pigmentation scores tend to have worse survival. Finally, a nomogram based on risk score, pigmentation score and other predictive factors were constructed. The AUC was 0.747 for two years, 0.737 for three years and 0.738 for five years, indicating well performance in terms of survival prediction.

Previous studies have well illustrated the roles of these four genes in tumor formation and progression. BOK (BCL-2–related ovarian killer), a member of the BCL-2 family, was firstly discovered by Hsu et al. (27) in 1997. Overexpression of BOK could induce cell apoptosis. Thus, delete of BOK was usually found in tumors (28), including breast cancer (29), non-small cell lung carcinoma (30) and colorectal cancer (31). In this current study, the result of q-PCR revealed that BOK was significantly down-regulated in melanoma tissues. However, we also noticed that increased BOK expression was linked to a poor prognosis, which was quite controversial. This controversial finding was also reported in colorectal cancer by Carberry et al. (31). They found that patients with stage II/III colorectal cancer showed a decreased level of BOK, but increased BOK level was associated with reduced overall survival time. The possible reasons for this result may be post-translationally regulations and intergenic interactions. Until now, no relevant studies have been reported in SKCM, and the roles of BOK in SKCM still need to be further explored. CD14 was the first recognized pattern recognition receptor that directly bands to LPS (lipopolysaccharide). It is a key factor in the TLR signaling pathway which has wide connections with inflammation and immune response. The relationship between CD14 and tumor formation is complex. On the one hand, CD14 could activate TLR-Myd88-NFκB and MyD88–CD14–IRAK1 pathway (32, 33) to promote tumor growth. On the other hand, activation of CD14 could also induce cell death and prevent tumorigenesis (34). Our study found that CD14 was highly expressed in SKCM samples but worked as a protective factor for SKCM patients. This result may be explained by the dual role of CD14 in tumor formation. Further in-depth studies are still in need. CYLD (CYLD lysine 63 deubiquitinase) regulates cell survival and proliferation (35). CYLD was reported as a tumor suppressor in SKCM (36–38). Our current study also found that CYLD was a down-regulated and protective factor. The expression pattern of CYLD was further validated in clinical samples by qRT-PCR. FASLG (also known as CD95L) is the natural ligand of FAS (also known as CD95). FAS could mediate apoptosis when bound to FASLG (39). However, it was reported that the expression of FASLG by apoptosis-resistant tumor cells could fight against antitumor cells. Thus, FASLG is found to be highly expressed in several tumors, including cervical cancer (40), melanoma (41) and bladder cancer (42). By using qRT-PCR, we also found that FASLG was highly expressed in melanoma samples. However, it seems to be a protective gene in the univariate Cox regression analysis. This could be explained by the pro-apoptosis function of FASLG. Further in-depth studies were still needed to explore the underlying mechanisms.

To further understand the mechanisms through which necroptosis-related genes may mediate differential prognosis in SKCM patients, we performed GO, KEGG, GSEA analysis and pigmentation phenotype analysis. The results indicated that necroptosis was strongly related to immune response and pigmentation, providing a foundation for further research into necroptosis processes.

Necroptosis is a form of programmed cell death that plays the role of a tumor suppressor in most tumors. The antitumor effect of necroptosis could partially be explained by the activation of the immune system (43). Firstly, necroptosis cells could release several inflammatory cytokines and chemokines (44). Production of cytokines could be directly induced by RIPK1 and RIPK3 which were key pro-necroptosis proteins (45, 46). Then, these cytokines could mediate cancer-relevant immune responses between dying cells and immune cells within the tumor microenvironment (47). Secondly, necroptotic cells could directly induce the activation of immune cells. Aaes et al. (48) noted that necroptosis in tumor cells is a form of immunogenic cell death, and necroptotic tumor cells could induce the proliferation of CD8^+^ cells. Similarly, other immune cells were also involved in the process of necroptosis, including natural killer cells (49), dendritic cells (50) and antigen-presenting cells (49). The results of our study were similar to previous studies. In KEGG analysis, the pathways were mainly enriched in cytokine-cytokine receptor interaction and chemokine signaling pathways. These findings were further validated in GSEA analysis. We also low-risk group patients showed highly enriched immune cells and highly activated immune-related pathways, indicating better survival of these patients might be related to necroptosis-activated immune responses.

Recent studies have shown that melanin pigmentation can affect the expression of many genes and many cellular processes of melanoma cells (17, 51). Thus, the forms of melanoma, including melanotic and amelanotic, should be considered during the evaluation of cell biology (16). Firstly, melanin pigmentation could affect the programmed cell death of melanoma cells. Cichorek et al. (16) reported that melanotic melanoma cells could resist camptothecin-induced apoptosis. Pawlikowska et al. (52) demonstrated that protein-bound polysaccharides (bioactive molecular obtained from Coriolus Versicolor) could induce necroptosis in amelanotic but not melanotic melanoma cells. These findings were similar to the conclusion of this current study. Patients from high-risk score groups tend to have higher pigmentation scores. In addition, positive correlations were found between risk score and pigmentation score. Although the identified four genes were not directly related to melanin pigmentation, the results still indicated the connection between melanin pigmentation and necroptosis. Secondly, melanin pigmentation could affect the survival of melanoma patients, partially because it could affect the therapeutic response (53–56). Brożyna et al. (57) reported that melanotic melanomas showed significantly shorter overall and disease-free survival. This trend was also found in this study. We also found that patients with high-risk and high pigmentation scores have even worse survival. Thirdly, melanin pigmentation can regulate the immune function of melanoma cells (52). Melanin could affect the activity of cytokine production and immune cells (58, 59), which were also found in our study.

ICIs, especially drugs targeting PD1 and CTLA4, has become a hot spot in cancer therapy in recent years. It was reported that ICIs could enhance the immune response triggered by necroptosis (43). In this current study, low-risk group patients showed better immunotherapy response when either PD1 or CTLA-4 was positively expressed. The following reasons could explain this. Firstly, the efficacy of ICIs was largely relying on immune functions (60). This current study shows that immune cell infiltration and expression of several checkpoint biomarkers were low in the high-risk score group. Secondly, melanin pigmentation could regulate the immune function of melanoma and thus affect immunotherapy (52). This study also indicates that pigmentation phenotypes should be considered during the treatment of melanoma patients.

This study also had many limitations. Firstly, more high-quality SKCM cohorts should be used to validate this prognostic model. Secondly, the underlying mechanisms of necroptosis still need to be further illustrated. Thirdly, the pigmentation phenotype of patients was evaluated by the GSVA analysis. Compared with Brożyna’s study which was based on the percentage of cells containing melanin, the accuracy of the GSVA method should be further validated. Despite these limitations, this is the first study that comprehensively analyzed the prognostic and immune implications of necroptosis in SKCM. More importantly, this study analyzed the melanin pigmentation phenotypes, an important feature of melanomas. This model was validated in an external cohort and clinical samples. Compared to similar studies, the results of this study were more convincing. This prognostic model may help clinical treatment and thereby improve patient outcomes.

## Conclusion

Finally, we developed a novel prognostic model based on the expression of four necroptosis-related genes, namely BOK, CD14, CYLD and FASLG. The risk score was an independent prognostic factor strongly related to patient prognosis. We hope this prognostic model can be used for individualized treatment for SKCM patients.

## Data Availability Statement

Publicly available datasets were analyzed in this study. These data can be found here: https://portal.gdc.cancer.gov/repository; http://www.ncbi.nlm.nih.gov/geo/; http://xena.ucsc.edu/. Further inquiries can be directed to the corresponding author.

## Ethics Statement

The studies involving human participants were reviewed and approved by the ethic committee of Chinese PLA General Hospital. The patients/participants provided their written informed consent to participate in this study.

## Author Contributions

Conceptualization, ZN and XW. Methodology, YX, YL, and XG. Data curation, YL, QZ, and BZ. Writing—original draft preparation, ZN, JX, and XP. Writing—review and editing, YH and WY. Supervision, YH and WY. All authors have read and agreed to the published version of the manuscript.

## Conflict of Interest

The authors declare that the research was conducted in the absence of any commercial or financial relationships that could be construed as a potential conflict of interest.

## Publisher’s Note

All claims expressed in this article are solely those of the authors and do not necessarily represent those of their affiliated organizations, or those of the publisher, the editors and the reviewers. Any product that may be evaluated in this article, or claim that may be made by its manufacturer, is not guaranteed or endorsed by the publisher.

## Supplementary Material

Supplementary Table 1 Primers used in qRT-PCR.
