# Supplementary material for: Development and Validation of a Novel Survival Model for Cutaneous Melanoma Based on Necroptosis-Related Genes
Source: Front Oncol. 2022 Mar 21;12:852803. doi: 10.3389/fonc.2022.852803 (PMC8979066; doi:10.3389/fonc.2022.852803)
Supplement: Supplementary Table 1 — Primers used in qRT-PCR. [file Table_1.docx]

| Name | Forward (5’-3′) | Reverse (5’-3′) |
| --- | --- | --- |
| BOK | GCGATGAGCTGGAGATGATCC | CTGCAGAGAAGATGTGGCCA |
| CD14 | ACGCCAGAACCTTGTGAGC | GCATGGATCTCCACCTCTACTG |
| CYLD | TCAGGCTTATGGAGCCAAGAA | ACTTCCCTTCGGTACTTTAAGGA |
| FASLG | ATTTAACAGGCAAGTCCAACTCA | GGCCACCCTTCTTATACTTCACT |
| GAPDH | GGAAGCTTGTCATCAATGGAAATC | TGATGACCCTTTTGGCTCCC |

Supplementary table 1: primers used in qRT-PCR.
